# Irish Travellers' Cancer Awareness and Engagement With Prevention, Screening and Early Diagnosis: A Qualitative Study of Barriers and Enablers

**DOI:** 10.1111/hex.70866

**Published:** 2026-09-21

**Authors:** Kate Frazer, Regina Joye, Patricia Fitzpatrick, Reuel Jalal, Mary Brigid Collins, Lynsey Kavanagh, Abigail Murfitt, Brigid Quirke, Una Kennedy, Triona McCarthy, Maria McEnery, Aine Lyng, Patricia Fox

**Affiliations:** ^1^ School of Nursing, Midwifery and Health Systems University College Dublin Dublin Ireland; ^2^ School of Public Health Physiotherapy and Sports Science (SPHPSS), UCD Dublin Ireland; ^3^ Pavee Point Traveller & Roma Centre, NGO Dublin Ireland; ^4^ National Social Inclusion Office, Health Service Executive Dublin Ireland; ^5^ National Cancer Control Programme, Health Service Executive Dublin Ireland

## Abstract

**Background:**

Irish Travellers are a distinct minority ethnic group with a long tradition of nomadism on the island of Ireland. Travellers have a significantly reduced life expectancy and experience greater health inequalities. Limited evidence describes how cancer awareness and engagement with prevention, screening and early diagnosis are shaped by structural and service contexts.

**Methods:**

This qualitative study was undertaken as part of a larger multi‐method study co‐developed with the Health Service Executive, National Cancer Control Programme, National Social Inclusion Office, and Pavee Point Traveller and Roma Centre‐ a human rights advocacy and Irish Non‐Governmental Organisation (NGO). The study aimed to understand cancer awareness and attitudes among Irish Travellers, identify barriers and enablers to cancer risk reduction behaviours and early diagnosis of cancer. In‐person interviews were completed with 22 adult Irish Travellers (18 individual interviews and two group interviews with two participants each) and were transcribed verbatim and analysed using collaborative reflexive thematic analysis.

**Results:**

Four themes described the interactions between everyday structural disadvantage, culture, privacy, fear and family roles; systems‐level barriers; and Traveller‐led and service‐level enablers. Awareness did not always translate into an opportunity to act. Racism and discrimination, literacy barriers, living conditions, limited service choice, inaccessible communication and inflexible appointment systems constrained engagement. Pride in Traveller identity, trusted relationships, Traveller Community Health Workers, accessible information and culturally safe services supported engagement.

**Conclusions:**

Improving cancer awareness requires more than individual behaviour change. Equitable engagement with prevention, screening and early diagnosis depends on anti‐racist, accessible and flexible mainstream services developed in partnership with Travellers and their organisations. Traveller Community Health Workers are a major strength, and their expertise should be harnessed within health systems.

**Patient and Public Contribution:**

The study was co‐developed with Pavee Point Traveller and Roma Centre, who are represented as co‐authors on the paper and were members of the study working group. They took part in all aspects of the study, including providing anti‐discrimination training, recruitment, interpretation of findings, and providing lived‐experience expertise throughout the drafting of the paper.

## 1 Background

Irish Travellers are a distinct minority ethnic group with a long tradition of nomadism on the island of Ireland [1, 2, 3, 4]. The All‐Ireland Traveller Health Study (AITHS) confirmed inequalities in health and the significant influence of the social determinants of health (SDoH) [1, 5]. Travellers experience markedly reduced life expectancy and increased premature mortality (standardised mortality ratio, 348; 95% CI, 298–397) [1, 5]. International evidence similarly identifies poorer health and access to care among Gypsy and Traveller populations than among other socially disadvantaged or minoritised groups, and calls for coordinated policy and services [6]. These inequalities are not explained by individual behaviour alone. Racism and discrimination within health and wider social systems, adverse effects on mental health, and structural barriers can reduce access to and engagement with health services [1, 3, 7, 8]. Cancer prevention and early diagnosis depend on access to understandable risk information, screening, timely primary care and follow‐up; however, minoritised ethnic groups may have lower awareness of cancer risk factors and reduced access to available services [9]. The Health Service Executive National Traveller Health Action Plan 2022–2027 provides a roadmap for targeted and mainstream service development in partnership with the Traveller community [4, 10].

Cancer awareness, therefore, needs to be considered alongside the conditions that determine whether people can understand, access and act on prevention, screening and early‐diagnosis opportunities. This qualitative component of a wider multi‐method study examined cancer awareness to assess risk reduction, screening and early diagnosis across cancer pathways. The analysis explored two connected questions: Travellers' cancer awareness and attitudes, and the structural, cultural and health‐system barriers and enablers that shape engagement. These data will inform targeted interventions, policy and service planning [10, 11].

## 2 Methods

### 2.1 Study Design

A qualitative study was undertaken as part of a larger multi‐method research project co‐developed with the Health Service Executive (HSE), National Cancer Control Programme (NCCP), National Social Inclusion Office (NSIO), and Pavee Point Traveller and Roma Centre (Pavee Point)‐ a human rights advocacy and Non‐Governmental Organisation (NGO). The Standards for Reporting Qualitative Research (SRQR) guided reporting (Appendix S1) [12].

### 2.2 Study Aim

The study aimed to explore (1) cancer awareness and attitudes among Irish Travellers and (2) the structural, cultural and health‐system barriers and enablers that shape cancer risk reduction, screening and early diagnosis.

### 2.3 Topic Guide

A topic guide was developed from current evidence and reviewed by the multidisciplinary study team and Traveller partners for relevance, clarity and cultural appropriateness (Appendix S2).

### 2.4 Recruitment

Recruitment was coordinated by Traveller Community Health Workers (TCHWs) working in Traveller Health Projects nationally and through Pavee Point. Traveller Community Health Workers are Travellers employed as peer health workers. Travellers who had participated in the national cancer awareness survey were invited to a subsequent in‐person interview. Contact details were shared with RJ with permission; RJ then contacted potential participants and arranged interviews in agreed, familiar locations used by TCHWs.

Inclusion criteria

- Adult Travellers aged 18 years and older
- Able to provide written/verbal consent
- Respondent of the Traveller survey

### 2.5 Data Collection

From November 2023 to February 2024, 22 participants took part in 18 individual interviews and two group interviews each comprising two participants. Group interviews were conducted at participants' request. Interviews lasted 13–49 min and were audio‐recorded with permission. Nineteen interviews were conducted by RJ; one was conducted jointly by RJ and PFo using the same topic guide, consent process and interview procedures. Participants were assured that data would remain confidential, reports would be anonymised, and no directly identifying information would be disclosed.

### 2.6 Analysis

Audio‐recordings were transcribed verbatim by a professional transcription company between January and February 2024 after identifying information was removed. Data were pseudo‐anonymised via an assigned code for identification. Braun and Clarke's six steps of Reflexive Thematic Analysis guided the process [13, 14]. Data familiarisation included reading the data and noting points of interest related to the research questions, specifically SDoH. Four members of the research team (PFo, RJo, AM, and KF) completed the analysis collaboratively through group familiarisation and in‐person meetings. The group compared interpretations, reflected on assumptions and initially coded semantic meaning before developing more latent interpretations through discussion. Codes were grouped into categories and subthemes, reviewed for overlap and refined into the four themes reported here. Differing interpretations were discussed reflexively rather than assessed through inter‐rater agreement. Following initial drafting, Traveller co‐authors from Pavee Point reviewed and refined the interpretation. This was co‐produced interpretive refinement, not participant member checking.

### 2.7 Positionality Statement

Analysis was completed by KF, PFo, and RJo, [academics and registered health care professionals], and AM, [postgraduate student] with experience in qualitative research and working with marginalised communities. RJ, a registered health care professional, completed interviews. The team considered how experience in professional roles, prior relationships, and assumptions could shape interpretation during reflexive group discussions. Pavee Point co‐authors contributed qualitative analysis expertise and lived experience with marginalised communities, providing additional qualitative and Traveller expertise and knowledge, including strengthening the lived experience and cultural interpretation of the findings.

## 3 Findings

### 3.1 Participant Characteristics

Twenty‐two Travellers participated: 16 women and 6 men (Table 1). Fifteen participants were aged 28–50 years, and 7 were older than 50 years; no men older than 50 years participated. Eighteen participants worked in Traveller organisations or health projects. Participants were recruited from six county locations; county‐level data are not presented to reduce the risk of deductive disclosure (Table 1).

**Table 1:** Demographic characteristics.

	Male	Female	Total *n*, %
**Location (County)**			
Dublin, Longford, Meath, Limerick, Cork, Sligo	6	16	22, 100%
**Age group**			
28–50 years	6	9	15 (68.2)
50 years +	0	7	7 (31.8)
**Total**	6	16	22 (100)

Four themes were developed (1) Life is Hard Enough; (2) Culture and Cultural Norms; (3) Systems‐level Barriers in Health Care, Cancer Prevention and Management: Wrong Words, Wrong Time; and (4) Achieving Self‐Determination in Health Care, Cancer Prevention and Management: An Equitable and Responsive Service. Across the themes, cancer awareness and opportunities to engage with prevention, screening and early diagnosis were shaped by the interaction of the social determinants of health (SDoH), racism, structural disadvantage, Traveller culture and the organisation of health services. The SDoH are the non‐medical factors and wider systems that shape lives, including economic policies, norms, and social and political systems that influence health and health equity [15, 16].

### 3.2 Theme 1: Life Is Hard Enough

This theme describes how structural disadvantage and everyday demands shaped cancer awareness and the capacity to act. Participants reported knowledge of symptoms and screening and described health‐promoting activities, but opportunities to use that knowledge were constrained by racism, discrimination, insecure or poor‐quality accommodation, poverty, education and literacy barriers, and cumulative stress. They also described difficult interactions with health professionals and long‐standing experiences of exclusion, sometimes beginning in childhood and contributing to intergenerational trauma. Some concealed their Traveller identity when seeking housing or employment.

Daily life required sustained attention to family and immediate needs. Women in particular described placing children and relatives before their own health. Delayed self‐care was therefore not simply a matter of low motivation: competing caring responsibilities, inadequate housing, poverty, stress and discrimination reduced the time, resources and emotional capacity available for cancer prevention. These social determinants compounded one another and affected both mental and physical health.

Living in areas you know with high noise pollution, living in areas beside pylons, electricity pylons which are quite prominent around Traveller sites actually. So I think there are environmental factors as well and financial factors. And I think also discrimination and racism, you know, I think you know, contribute to ill health in many ways. Mental health issues, physical health issues. (Participant 2)…. there is not a lot of information there like with leaflets it's not Traveller appropriate, it's not how important it is to a Traveller to look after their own health. To change the statistics from 66 for a woman to die, to change it to 76, do you know what I mean….

(Participant 5)

Participants described unequal educational opportunities and literacy barriers, particularly among some older Travellers. Difficulties arose when health information was delivered through complex written materials, appointment letters or postal systems that did not reflect people's communication needs or living circumstances. These barriers could lead to misunderstanding and missed appointments.

…unfortunately, there are many other Travellers out there who cannot read and write very well. So, a lot of the material is not very accessible in the mainstream.

(Participant 2)

So I would neglect my health and make sure that they're okay. And that's where all of these things. There's such a connection with education, poverty. and your education is good.

(Participant 8)

Cancer‐related choices were made within conditions of persistent poverty, limited employment and educational opportunities, racism and stigma. Participants discussed smoking, alcohol, vaping and sunbed use, but also identified factors that improved cancer awareness: literacy support, contact with Traveller health projects, supportive health professionals, trusted general practitioners (GPs), Traveller organisations and accessible social‐media information. When families were managing food, heating, housing or acute stress, cancer prevention could become a lower immediate priority.

We can say housing, but the poor housing, poor accommodation, contributes to so many factors and the result of their health. It has grown this long, as I say it's grown as long as the county of [xx] it's the environment. And this is why Traveller people are dying a lot younger, and the mental health has deteriorated to the level that it has. Like this morning one of the girls came in and said did you know that alcohol has an effect on cancer, I'm saying sometimes you want to have the alcohol to get over what's going on in your head but then you wake up and you lose the next day, and you are still not any better. Because you still have to deal with what's in your head.

(Participant 21)

Well, the screening tests for cancer it's very, very, none at all. Because I don't think the Travellers is aware, I'm aware of screening tests but there's a lot of the Travellers is not, not aware of it. No awareness of it; again, as I said, it has to go out there again. More education out there. It's all coming back to education the whole time.

(Participant 15)

These findings distinguish awareness from opportunity. Knowledge varied within the community, but the capacity to act on symptoms, prevention or screening depended on resources, trusted support and accessible services. The enabling conditions are developed further in Theme 4.

### 3.3 Theme 2: Culture and Cultural Norms

This theme explores cultural meanings that participants considered relevant to cancer awareness and engagement, including pride and belonging, family roles, privacy and confidentiality, religious coping, fear, fatalism and gendered help‐seeking. Travellers are a heterogeneous community; these meanings were neither universal nor isolated from structural exclusion. Participants described culture as interacting with previous experiences of discrimination, limited service choice and the organisation of healthcare.

Many participants expressed pride in being Travellers and a strong sense of belonging to an extended family and community. Some described prayer, pilgrimage and religious faith as important responses when cancer evoked fear or bereavement. These accounts should not be interpreted as a uniform rejection of medical care. Rather, spiritual coping, fear, fatalism and delayed help‐seeking were described in the context of painful family experiences, social exclusion and uneven trust in or access to services.

The Travellers in their tradition or whatever would be like that they would be looking for prayers, for people to pray for them that they have cancer and that they hopefully will get cured.

(Participant 6)

Privacy and confidentiality were central concerns. Limited choice of GP meant that several Traveller families could attend the same practice, creating fears of being recognised, overheard or discussed. Participants also described reluctance to disclose sensitive information to reception or administrative staff by telephone or in a shared waiting area. A trusted and welcoming practice could improve access, while the concentration of patients within a small number of practices could simultaneously reduce perceived privacy.

Travellers, in general, are very private about their health. So, I would say that they know a good bit, but they're not saying that they actually. Like it wouldn't be, like you'd hear of Travellers, unfortunately, you'll hear of Travellers when they have the cancer, you wouldn't know, or it wouldn't be common knowledge that they're going, that they're attending the clinics; until they actually maybe could be waiting for treatment. That they actually have been told 100% that they have cancer and that they're going to get treatment.

(Participant 6)

See, the stigma is there with Travellers; it's often shame, they're ashamed if they go to a service. In case they meet somebody in there. And Travellers are very confidential. If they are diagnosed with cancer, they're not going to tell people straight away. They kind of keep it to the one family.

(Participants 12 and 13)

There's too many Travellers for the one doctor. Because they can't get no one else so one doctor takes a lot of Travellers on. But sometimes a lot of Travellers want a bit or privacy or whatever. So, some Travellers are afraid sometimes that this doctor is going to discuss your problems with them or if the receptionist at the desk is talking on the phone, you're kind of afraid to talk to her because there might be someone in the waiting room.

(Participant 1)

Some participants described fear and fatalistic beliefs that could discourage engagement with cancer services or early diagnosis. Cancer was sometimes associated with death, bad luck or inevitability, which could foster avoidance or a sense of limited control. These accounts were not presented as fixed characteristics of Traveller culture; they interacted with previous bereavement, exclusion and limited access to understandable, trusted information. Participants contrasted these barriers with the culturally informed listening and relationships provided by TCHWs.

…. there's a lot of fear there. A lot of Travellers, I think some Travellers wouldn't want to know. Just that fear around the word cancer is huge in the Traveller community. So, they kind of turn a blind eye to it.

(Participant 1)

Some Travellers out there that won't even say the word cancer. Because they are afraid of what it might bring and what it might implement. It's terrifying, and personally myself it would be something I'd be terrified of because it's been in my family so much, unfortunately.

(Participant 18)

A lot of the Travellers and especially the older Travellers when they find out about cancer, that they have cancer or that a member of the family has cancer, they do, they turn down the religious road. They go to all these pilgrims, and they could be a different country, Medjugorje or Lourdes, all of these places. They go to all the priests around the country, they'll go to all these places and to me this is burying their heads in the sand.

(Participant 7)

Family, including extended relatives, was highly important. Participants described euphemisms such as ‘women's problems’ when cancer was difficult to name and linked secrecy to fear, embarrassment and painful family memories. They also described gendered help‐seeking: women frequently organised appointments and encouraged men to seek care or attend screening. These accounts suggest that gender roles may influence engagement, although the absence of men older than 50 years limits interpretation of older men's experiences.

If a woman had cancer there, she wouldn't tell it. The Traveller woman wouldn't tell it because they'd be too embarrassed for to tell it. ……. It's very hard to get a man to, because fear, like they're big strong men like but it's, the women has to do it all for them, do you know make their appointment and try and get them to it. Because they don't want to go to the doctors. And it's like that they're burying their head. …. There is men, more men than women, that's afraid to go and get their selves checked.

(Participant 7)

### 3.4 Theme 3: Systems‐Level Barriers in Health Care, Cancer Prevention and Management: Wrong Words, Wrong Time

This theme describes barriers created by the organisation of health services and their interaction with living conditions, literacy, transport, caring responsibilities and discrimination. Participants wanted to report symptoms and use services, but described a demanding journey through primary and secondary care. Pressures such as delayed appointments or rushed consultations may affect the wider population, but their consequences were intensified for Travellers by limited service choice, inaccessible communication and difficulty navigating systems.

Participants described delays in obtaining appointments, inflexible booking and rescheduling processes, postal and digital communication, limited transport, insufficient gender‐appropriate care, rushed consultations and fragmented pathways. They also referred to the unequal access created by the two‐tier Irish health system. Together, these barriers could lead to withdrawal from services and the conclusion that attending was ‘not worth the hassle'.

And you might make a phone call, and you mightn't get an appointment for a week or two…It is a common problem when they go to doctors. They're coming out no wiser like. Because, like, they could go to a doctor and they come out a lot worse because they've been given all those big wordings and things. And names and things. …. So maybe some doctors wouldn't, you might have a doctor who might have a great time for a person and explain everything properly. But then you might have a doctor that just wants you out faster……Just rush. Rush you. …… That you're in there for maybe a minute or two and they just want you in and out fast. You tell them your symptoms, and they just can't wait to get you out. So, I think a doctor should give that Traveller person more time. Because over the lack of education, they have. And do more clearer words for them. Give them a better understanding.

(Participants 12, 13)

Caring responsibilities and appointment systems interacted. Traveller women, like many mothers, described prioritising family and being unable to attend appointments that conflicted with childcare or school collection. Postal delays, inaccessible letters and uncertainty about how to rearrange appointments increased the risk of non‐attendance. Rather than assuming a lack of responsibility, participants' accounts show that mainstream systems often failed to explain consequences, offer accessible alternatives or support people to navigate the pathway.

…. You just put it off like that. And a lot of Traveller women, I think women in general they put themselves last, they put like the family would be first or the children would be first …. Like if the appointment just say was at 2 o'clock and they'd to collect their children at 2 o'clock, well then they wouldn't go to the appointment.

(Participant 6)

The quotations illustrate how routine service processes can become structural barriers. Leaving a consultation ‘no wiser’ or ‘a lot worse’ reflected inaccessible terminology, insufficient time and a failure to check understanding. Transport, post and app‐based booking further reduced access. These findings lead into Theme 4, which identifies the trusted relationships, communication practices and service adaptations that participants believed could support engagement.

….A lot of sites where Travellers live, there's no real public transport, so if you're not driving you're kind of, you're in a bit of trouble there because you probably have to walk to the doctors sometimes and then you're kind of saying to yourself look it's not worth the hassle. I won't even bother going to the appointment today. And then you have the issue of posts, the postmen or women delivering post to the sites, you mightn't get the post for weeks and then, or you mightn't get the post for a few days, and then when you do get it, you realise that you've missed an appointment. So that's another struggle that we have, that Travellers have. …. And then also this new thing now, the doctors are doing, where sometimes you've to download an app to book an appointment. A lot of Travellers wouldn't have a clue how to; my own parents, my father especially he only has the normal, they wouldn't have a phone, a smartphone. He just has a phone that has numbers on it. And he can't check into that or whatever.

(Participant 1)

I suppose when people do receive appointments because some people, sometimes it can be very hard to understand appointments as well when they're sent out to people. Maybe a person could be waiting a couple of months; it could be a year down the line. They get this appointment from, it could be from the hospital, it could be from somewhere. And they're just thinking what is it for and it's not explained enough in the appointment what the appointment is for, do you understand……. an awful lot of Travellers I think misses their appointments. But if they do miss the appointment, they've got to understand they need to phone up and tell them they can't make it. Instead of not contacting them. Do you know like some people they've an appointment today, and they can't make it. Well, you should phone up the hospital whatever you'd be doing and say you want an appointment for a later date. Because if you don't ring them, they're not going to sent another appointment out to you. It's giving Travellers a better understanding on the way to manage their health like. Especially getting appointments.

(Participants 12,13)

### 3.5 Theme 4: Achieving Self‐Determination in Health Care, Cancer Prevention and Management: An Equitable and Responsive Service

This final theme foregrounds enabling conditions for cancer awareness, prevention, screening and early diagnosis. Participants identified Traveller‐led health projects, trusted relationships, accessible information, gender‐appropriate care and respectful professionals as important strengths. TCHWs combined cultural expertise, local knowledge and sustained relationships, enabling them to translate information, support navigation and create psychologically safe spaces.

Yeah, the Primary Health Care workers have a very important role; they go out to the homes and advise people of all different things, like healthy eating, smoking groups, nonsmoking groups and all that, advising people that wouldn't get out. They actually go to their homes and tell them about it…. sometimes, the Primary Health Care workers here call out to the homes, and they invite people for breast checks and screening and all that. And they kind of talk them into going to it. So usually they always usually go. So that's a very good help to people that doesn't, like, refuses appointments and stuff.

(Participant 11)

Participants valued direct engagement with healthcare professionals, especially public health nurses and others who attended Traveller Health Projects in familiar, non‐intimidating settings. They also valued materials developed with TCHWs, plain‐language communication, time to listen and opportunities to ask questions. These strengths could be embedded more consistently through formal partnership between Traveller projects and mainstream services, rather than depending on isolated examples of good practice.

… Like in the kind of leaflets we would build up. Like we would make sure that they'd understand what would be on it. So, in other words, understanding as well can be a big thing for Travellers when it comes to certain things like…. I think it could yeah, have some kind of workshops or something on those awareness. Would help bring awareness there to people.

(Participants 12, 13)

Well, cancer screening, …… more Traveller friendly spaces for it, you know, surgeries or cancer screening services. And be always totally aware of the literacy but also be aware of the culture of cancer screening or the culture of our community regarding women and screening. And maybe this is in the wider community as well; Traveller women would tend to want a female health people to do the test on them rather than having males to do them. So, you know just to be are of all that, be aware of the culture regarding that and be aware of the literacy definitely.

(Participant 16)

Participants proposed multiple routes for improving awareness and screening, including workshops, schools, sports clubs, informal conversations, trusted digital channels and partnerships with cancer charities. These approaches draw on community strengths, but TCHWs cannot be the sole bridge into healthcare. Mainstream cancer and health services retain responsibility for providing confidential, anti‐racist, accessible and culturally safe care.

I think the Primary Health Care workers really do fantastic work in producing information and material and resources that is very easy to understand and very accessible. And I think that's really important…. You know, as time goes on, you know, and I think the next generation after this generation will probably be even more enlightened. And I think that's just a natural order of things really in my opinion. But the older generation, if you like, they will tell you that most of their information actually comes directly from Traveller organisations. Not from mass media, not from the health care sector, but from Traveller organisations, you know, producing, as I said earlier on, very accessible, easy to read, easy to understand material around health issues, you know, whether that's cardiovascular disease, cancer, diabetes, whatever else it might be. So I think that's where we're at.

(Participant 2)

We have a lot of Primary Health Care workers that work with Traveller families out in the local areas. And I would get the information from them. But also probably from my sports clubs or if I'm involved in sport. I get it from my trainers or whatever about healthy living, healthy eating, all that kind of stuff.

(Participant 1)

Collectively, the enablers show how awareness can be converted into an opportunity to act: information must be understandable, services must be reachable and trustworthy, and Traveller expertise must be resourced and integrated into, rather than substituted for, mainstream provision.

## 4 Discussion

The principal finding is that Travellers' cancer awareness and opportunities to engage with prevention, screening and early diagnosis are shaped by the interaction of structural disadvantage, cultural meanings and the organisation of health services. Awareness and attitudes, barriers and enablers, are therefore connected rather than separate study aims. Participants could recognise symptoms, risk factors or screening opportunities while still being unable to act because of racism, poverty, literacy barriers, caring demands, limited service choice and inaccessible systems [1, 2, 8, 9, 11, 17].

This distinction between awareness and opportunity helps explain why increasing knowledge alone is unlikely to reduce inequity. The National Cancer Strategy seeks to improve awareness, screening and early diagnosis, but these goals cannot be realised for Travellers without action on the wider conditions that shape access and trust [7, 8, 9, 10, 11]. Irish and international evidence consistently identifies discrimination and exclusion across housing, education and services, with consequences for health and engagement [8, 18, 19, 20]. Socioeconomic conditions may also contribute to intergenerational patterns of disadvantage, with parental adverse childhood experiences associated with subsequent healthcare use [21]. The current findings extend this evidence by showing how all these conditions enter cancer pathways through communication, appointments, transport, continuity and the capacity to prioritise preventive care.

Cultural meanings also require contextual interpretation. Pride in Traveller identity and strong family relationships were sources of support, while privacy, fear, religious coping, fatalistic beliefs and gendered roles influenced how cancer was discussed and when help was sought. These should not be treated as self‐contained cultural deficits. They interacted with bereavement, previous discrimination, limited privacy and uneven access to trusted medical care, consistent with participatory research with Gypsy, Roma and Traveller communities [9, 22, 23, 24]. Spiritual support and evidence‐based cancer care need not be mutually exclusive; services should provide non‐judgmental information while respecting people's beliefs and autonomy. Participants also described health‐system pressures experienced by many people, including rushed consultations, delayed appointments, childcare conflicts, and complex referral processes, but these effects were amplified by literacy barriers, transport difficulties, postal delays, digital exclusion, and a restricted choice of GP. The quotation about leaving a consultation ‘no wiser’ and ‘a lot worse’ demonstrates the clinical consequences of communication that does not check understanding. The TCHWs and Traveller organisations help bridge these gaps and support screening engagement, but reliance on them alone risks leaving mainstream services unchanged [25].

The National Traveller Health Action Plan supports partnership, integration and culturally responsive provision across the health system [10]. Participants' accounts of smoking, alcohol, vaping and sunbed use also place cancer risk behaviour within commercial and social environments rather than individual choice alone. Access by minors to sunbeds and changes in the legal age for tobacco and nicotine products illustrate the importance of regulation and commercial determinants alongside education [26, 27].

Table 2 translates the findings into paired barriers and enabling conditions. Priorities include ongoing anti‐racism work, plain‐language and multimodal communication, flexible appointments, confidential pathways, gender‐appropriate services, sustained investment in TCHWs and formal collaboration between Traveller organisations and mainstream cancer services.

**Table 2:** Summary of Barriers and Enabling Conditions for Cancer Awareness and Engagement Identified by Irish Travellers.

Structural and social context	Cancer‐awareness and service barriers identified	Enabling conditions identified
Racism and discrimination	Previous discriminatory encounters; distrust; reduced service access; concealment of identity	Ongoing anti‐racism training and accountability; respectful, culturally safe care
Education and health literacy	Complex terminology; inaccessible letters and materials; online‐only processes; difficulty understanding appointments	Plain‐language, visual and verbal information; multiple communication formats; time to check understanding
Service access and organisation	Few accessible GPs; delays; rushed consultations; fragmented care; inflexible booking and rescheduling	Trusted professionals; flexible appointments and rescheduling; integrated, person‐centred care
Poverty, living and environmental conditions	Transport and postal barriers; competing costs; insecure or poor accommodation; immediate survival priorities	Outreach, transport and navigation support; flexible contact methods; coordination with local Traveller projects
Privacy and confidentiality	Limited provider choice; several families using the same practice; disclosure to administrative staff or being overheard	Private communication routes; explicit confidentiality assurances; choice of professional or service where possible
Family and gender roles	Caring responsibilities; appointments conflicting with childcare; reluctance among some men; limited gender‐appropriate care	Gender‐appropriate services; flexible timing; women and men Traveller health workers; family‐sensitive approaches
Fear, cancer beliefs and religious coping	Cancer associated with death; fear or fatalism; delayed help‐seeking	Non‐judgmental conversations; early‐diagnosis messages; spiritual support alongside evidence‐based care
Community infrastructure and TCHWs	Too few workers; insecure funding; limited integration with mainstream services	Sustained funding and upskilling; formal partnerships; TCHW roles linked with mainstream services
Information channels	Mainstream resources not Traveller‐appropriate; limited awareness of screening and services	Traveller‐led materials; informal conversations; workshops; schools, sports and trusted digital channels

## 5 Limitations

Recruitment was challenging and required an ethics amendment to widen opportunities. Recruitment through Traveller organisations and health projects meant that most participants were connected to these supports; 18 worked in Traveller organisations or projects and may have had greater exposure to health information than Travellers with smaller networks or less access to community infrastructure. The sample was predominantly female, and no men older than 50 years participated, limiting interpretation of older men's awareness and help‐seeking. The qualitative sample was not intended to be statistically representative, and two group interviews may have influenced what participants chose to disclose. Pavee Point co‐authors contributed interpretive refinement, but formal participant member checking was not undertaken. Recruitment also coincided with religious and family holiday periods and with bereavements from cancer, when engagement through gatekeepers was neither appropriate nor possible. Finally, the timing of the national survey meant that survey findings could not inform the topic guide as originally planned. These contextual limitations should be considered when assessing transferability.

## 6 Conclusion

Travellers' cancer awareness and engagement with prevention, screening and early diagnosis are shaped by the interaction of structural disadvantage, Traveller culture and the organisation of health services. Increasing knowledge alone is insufficient where racism, poverty, literacy barriers, privacy concerns and inflexible systems constrain the opportunity to act. Mainstream services should deliver anti‐racist, confidential care, accessible communication, flexible appointment pathways, and gender‐appropriate services. Traveller organisations and TCHWs are trusted strengths that should be sustainably resourced and integrated into health services via workforce developments, but they cannot be the only route to equitable care. Partnership with Traveller organisations can translate into practical opportunities for prevention and early diagnosis, helping reduce inequities in cancer outcomes.

## Author Contributions

**Kate Frazer:** conceptualization, investigation, funding acquisition, writing – original draft, methodology, validation, writing – review and editing, formal analysis, project administration. **Regina Joye:** conceptualization, investigation, funding acquisition, writing – original draft, methodology, writing – review and editing, formal analysis, project administration, validation. **Patricia Fitzpatrick:** conceptualization, investigation, funding acquisition, methodology, writing – review and editing, project administration. **Reuel Jalal:** writing – review and editing, data curation, project administration, methodology. **Mary Brigid Collins:** methodology, writing – review and editing, formal analysis, data curation, resources, conceptualization. **Lynsey Kavanagh:** methodology, writing – review and editing, formal analysis, data curation, conceptualization. **Abigail Murfitt:** writing – review and editing, formal analysis. **Brigid Quirke:** writing – review and editing, methodology, conceptualization. **Una Kennedy:** conceptualization, writing – review and editing. **Triona McCarthy:** conceptualization, writing – review and editing. **Maria McEnery:** conceptualization, writing – review and editing. **Aine Lyng:** conceptualization, writing – review and editing. **Patricia Fox:** conceptualization, investigation, funding acquisition, writing – original draft, methodology, validation, writing – review and editing, formal analysis, data curation, project administration.

## Ethics Statement

Ethical approval was obtained from (1) Pavee Point Research Advisory Committee and (2) Royal College of Physicians of Ireland (RCPI RECSAF 191, May 2023, amended December 2023). A university Research Ethics Committee reviewed and granted a low‐risk approval. Participants' consent to participate in the study included permission to publish the findings of the research.

## Conflicts of Interest

The authors declare no conflicts of interest.

## Supporting information


Supporting File 1

Supporting File 2

Supporting File 3

## Data Availability

The data that support the findings of this study are not publicly available and cannot be shared due to privacy or ethical restrictions. Enquiries regarding the data may be directed to the corresponding author.

## References

[1] All Ireland Traveller Health Study: Our Geels (2010), available from: https://www.ucd.ie/t4cms/AITHS_SUMMARY.pdf.

[2] B. Quirke , M. Heinen , P. Fitzpatrick , S. McKey , K. M. Malone , and C. Kelleher , “Experience of Discrimination and Engagement With Mental Health and Other Services by Travellers in Ireland: Findings from the All Ireland Traveller Health Study (AITHS),” Irish Journal of Psychological Medicine 39, no. 2 (2022): 185–195, 10.1017/ipm.2020.90. 32847636

[3] F. Kennedy , A. Ward , D. Mockler , J. Villani , and J. Broderick , “Scoping Review on Physical Health Conditions in Irish Travellers (Mincéiri),” BMJ Open 13, no. 8 (2023): e068876. 10.1136/bmjopen-2022-068876 PMC10462949 37640559

[4] Central Statistics Office , Census 2022 Profile 5 ‐ Diversity, Migration, Ethnicity, Irish Travellers & Religion (2023), accessed November 20, 2025, available from: https://www.cso.ie/en/releasesandpublications/ep/p‐cpp5/census2022profile5‐diversitymigrationethnicityirishtravellersreligion/irishtravellers/.

[5] S. Abdalla , C. Kelleher , B. Quirke , L. Daly , and All‐Ireland Traveller Health Study team , “Social Inequalities in Health Expectancy and the Contribution of Mortality and Morbidity: The Case of Irish Travellers,” Journal of Public Health 35, no. 4 (2013): 533–540. 10.1093/pubmed/fds106 23315684

[6] G. Parry , P. Van Cleemput , J. Peters , S. Walters , K. Thomas , and C. Cooper , “Health Status of Gypsies and Travellers in England,” Journal of Epidemiology & Community Health 61, no. 3 (2007): 198–204. 10.1136/jech.2006.045997 PMC2652907 17325395

[7] C. McGorrian , K. Frazer , L. Daly , and All‐Ireland Traveller Health Study Research Team , “The Health Care Experiences of Travellers Compared to the General Population: The All‐Ireland Traveller Health Study,” Journal of Health Services Research & Policy 17, no. 3 (2012): 173–180, 10.1258/JHSRP.2011.011079. 22761349

[8] E. Carron‐Kee , F. McGinnity , and A. Alamir , “Understanding Attitudes to Travellers and Roma in Ireland,” Economic and Social Research Institute (ESRI) Research Series 1 (2024): 10.

[9] L. Condon , J. Curejova , D. L. Morgan , G. Miles , and D. Fenlon , “Knowledge and Experience of Cancer Prevention and Screening among Gypsies, Roma and Travellers: A Participatory Qualitative Study,” BMC Public Health 21, no. 1 (2021): 360. 10.1186/s12889-021-10390-y PMC7885498 33593323

[10] Health Service Executive , National Traveller Health Action Plan 2022–2027 (Health Service Executive, 2022).

[11] Department of Health , National Cancer Strategy: 2017–2026 (Department of Health, 2017), available at: https://assets.gov.ie/9315/6f1592a09583421baa87de3a7e9cb619.pdf.

[12] B. C. O'Brien , I. B. Harris , T. J. Beckman , D. A. Reed , and D. A. Cook , “The SRQR Reporting Checklist,” in The EQUATOR Network Reporting Guideline Platform [Internet], eds. J. Harwood , C. Albury , J. de Beyer , M. Schlüssel , and G. Collins (The UK EQUATOR Centre, (2025), available from: https://resources.equator‐network.org/reporting‐guidelines/srqr/srqr‐checklist.docx.

[13] V. Braun and V. Clarke , Thematic Analysis: A Practical Guide (Sage Publishing, 2021).

[14] V. Braun and V. Clarke , “A Critical Review of the Reporting of Reflexive Thematic Analysis in Health Promotion International,” Health Promotion International 39, no. 3 (2024): daae049. 10.1093/heapro/daae049 PMC11132294 38805676

[15] World Health Organisation , Social Determinants of Health, Web page (2024), accessed September 17, 2024, https://www.who.int/health‐topics/social‐determinants‐of‐health#tab=tab_1.

[16] M. Marmot , “‘Social Determinants of Health Inequalities’,” Lancet 365, no. 9464 (2005): 1099–1104, available at: 10.1016/S0140-6736(05)71146-6. 15781105

[17] J. Villani and M. M. Barry , “A Qualitative Study of the Perceptions of Mental Health among the Traveller Community in Ireland,” Health Promotion International 36, no. 5 (2021): 1450–1462. 10.1093/heapro/daab009 33582793

[18] Irish Human Rights and Equality Commission (IHREC) , Membership of the Traveller Community (2024), accessed November 25, 2025, https://www.ihrec.ie/your‐rights/services/membership‐of‐the‐traveller‐community/.

[19] J. M. Cénat , “Racial Discrimination in Healthcare Services among Black Individuals in Canada as a Major Threat for Public Health: Its Association With COVID‐19 Vaccine Mistrust and Uptake, Conspiracy Beliefs, Depression, Anxiety, Stress, and Community Resilience,” Public Health 230 (2024): 207–215. 10.1016/j.puhe.2024.02.030 38574426

[20] L. E. Egede , R. J. Walker , and J. S. Williams , “Addressing Structural Inequalities, Structural Racism, and Social Determinants of Health: A Vision for the Future,” Journal of General Internal Medicine 39, no. 3 (2024): 487–491, 10.1007/s11606-023-08426-7. PMC10897090 37740168

[21] M. F. Ketelaars , M. Sprenger , A. J. Bos , et al., “The Impact of Parental Adverse Childhood Experiences on Children's Healthcare Utilisation: A Systematic Review,” medRxiv 1 (2025): 25328669. 10.1136/jech-2025-224640 PMC12911568 41219060

[22] N. Buckley , Gypsies' and Travellers' Lived Experiences, Culture and Identities, England and Wales: 2022: Qualitative Research Exploring the Lived Experiences of Gypsy and Traveller communities, Relating to Culture and Identities (2022), accessed November 25, 2025, https://www.ons.gov.uk/peoplepopulationandcommunity/culturalidentity/ethnicity/bulletins/gypsiesandtravellerslivedexperiencescultureandidentityenglandandwales/2022.

[23] E. Speed, Building a Community of Practice to Identify Strengths, Barriers and Prioritise Solutions to the Right of Access to Healthcare for Travelling Communities, accessed November 25, 2025, https://arc‐eoe.nihr.ac.uk/news‐blogs/news‐latest/research‐highlights‐challenges‐and‐pathways‐better‐healthcare‐gypsy‐roma‐and.

[24] L. Condon , J. Curejova , D. Leeanne Morgan , and D. Fenlon , “Cancer Diagnosis, Treatment and Care: A Qualitative Study of the Experiences and Health Service Use of Roma, Gypsies and Travellers,” European Journal of Cancer Care 30, no. 5 (2021): e13439. 10.1111/ecc.13439 33955101

[25] P. Fitzpatrick , A. O'Flynn , R. Jalal , et al., “Identifying Barriers and Facilitators to Participation in Cancer Screening Among Irish Travellers, a Minority Ethnic Group in Ireland, Using a Codesigned Approach,” Journal of Medical Screening 33 (2025): 9–14. 10.1177/09691413251352402 40567151

[26] G. Deegan , “Judge Expresses Concern that Sunbeds Are Still Allowed for Adults Amid Illegal Use by Persons Under 18,” *Irish Times* May 10, 2025, https://www.irishtimes.com/crime‐law/courts/2025/05/10/judge‐expresses‐concern‐that‐sunbeds‐are‐still‐allowed‐for‐adults‐amid‐illegal‐use‐by‐person‐under‐18/.

[27] Government of Ireland , “Government Approves Legislation to Increase the Minimum Legal Age of Sale of Tobacco Products to 21,” *Press Release*, 2024, https://www.gov.ie/en/department‐of‐health/press‐releases/government‐approves‐legislation‐to‐increase‐the‐minimum‐legal‐age‐of‐sale‐of‐tobacco‐products‐to‐21/.

