# Supplementary material for: Irish Travellers' Cancer Awareness and Engagement With Prevention, Screening and Early Diagnosis: A Qualitative Study of Barriers and Enablers
Source: Health Expect. 2026 Sep 21;29(5):e70866. doi: 10.1111/hex.70866 (PMC13594775; doi:10.1111/hex.70866)
Supplement: Supplementary file 1 [file HEX-29-e70866-s001.pdf]

Semi-structured Interviews/Focus Group with Travellers
Topic Guide

Logos removed for Peer Review

Cancer Awareness and Attitudes among the Traveller Community in Ireland

Semi-structured Interviews/Focus Groups with Travellers
Topic Guide

Following Receipt of Informed Consent

Thank you very much for completing the survey and for agreeing to this follow-up interview. We really appreciate you taking the time to take part in this research study.

Some of the questions I am going to ask here might sound like the questions asked in the survey, but in the interviews, we are hoping to understand your views in a little more detail. Again, just to reassure you, I will not mention your name once the recording starts and your name will not be included anywhere in the final report.

There are no right or wrong answers, it is just your views and experience we are interested in with regard to reducing the risk of cancer and accessing services. Please let me know if I am not being clear at any point and I will be happy to repeat the question. Some of the questions, you may not know the answer to and that is absolutely fine.

Request permission to request age and gender

Where do you usually get information on how to be healthy to reduce your risk of cancer…. who have you spoken to… can you please tell me what happened?
And for Travellers' as a community, can you let me know where would they get their information … who would they normally speak to?
Prompts, if needed) what about the doctor, nurse Traveller primary health care workers

What do you think stops Travellers' from finding out about things that are known to cause cancer?
Prompts, if needed)
What about things like getting to see a doctor or nurse? Getting an appointment; Transport or the lack of it?
What about education … being able to read?
And what about housing conditions? Not getting post?

Any other things?

In your experience, how likely are Travellers to start and continue to do things that can reduce the risk of getting cancer? For example, stopping smoking or not smoking, not drinking alcohol, trying to exercise most days, eating healthily.

If the answer is unlikely: Why do you think that is?
If not clear to the participant: What do you think stops Travellers from starting or continuing to do things that reduce the risk of getting cancer?
(Can use prompts if needed ….
Housing conditions: not having room or facilities to cook healthy food?
Living in an area where there are no parks/safe areas to exercise?
Not being able to get an appointment with a GP to get help with stopping smoking?
Unemployment: not being able to afford healthy food? Sunscreen?
Transport: not being able to attend stop smoking education,transport, times of clinics)
Education: related to use of sunbeds?

And what do you think would help Travellers to start or continue to do things that reduce the risk of getting cancer?
(Can use prompts if needed …. Traveller Primary Healthcare Workers, primary care health projects, housing, employment, education, transport, times of clinics)

Thank you, I'd now like to ask you some questions about cancer screening ……(as applicable)
Prompts, if needed) Screening are tests done to identify cancer early in the population (in people who do not have symptoms of cancer) e.g, cervical smear tests, or breast check x-ray tests of the breast called mammograms.
What do you know about cancer screening in Ireland?
Can you tell me about your experiences or what you've heard from your family and the other members of the Traveller community?
…. experiences of stories about screening
for breast cancer ….
for cervical cancer - smear test/new HPV test
for bowel cancer…

If the participant has participated in screening:
What helped you to take part in the cancer screening programmes?
In your experience, what has helped other Travellers to take part in cancer screening programmes?

What do you think stops Travellers' from taking part in cancer screening?

What else do you think would help Travellers' to take part…. what do health services like the HSE need to do?
Some cancer screening programmes send information and tests in the post….. Do you know about this?
How does that work for the Traveller community … can you tell me please what you have heard or know about these tests?
There's a plan to send another kit in the post for cervical cancer screening - this would allow women to take their own cervical screening test, using a thing like a long cotton bud which would then be sent back for testing. So the person would not have to go to the GP for the test.
Would that be something that might help?….. how would that work for the Traveller community …..

What other suggestions do you have……..

Thank you; I now have a few questions to ask about cancer awareness.

In your experience, how much do Travellers' know about the signs and symptoms of cancer …. (prompts of unusual bleeding, hoarseness, losing a lot of weight when not trying to lose weight, etc).
Who would Travellers generally talk to about any of these symptoms?
(prompt if needed - Traveller Primary Healthcare Worker, doctor, nurse)
What is most helpful in this situation (prompt if needed - Traveller Primary Healthcare Worker, doctor, nurse …..)
What else do you think would be helpful for Travellers with regard to reporting symptoms they are worried about?

If response is that symptoms are not reported early,
What do you think stops Travellers' from seeking help (from their GP, PHN or in casualty) if they are worried about a symptom that might be related to cancer?
What else do you think might stop Travellers' from getting help early?
Prompt if needed … What about fear, past experiences of cancer in the family?
What about lack of transport, arranging an appointment for a doctor or nurse?
What about housing/dealing with stress/issues such as homelessness/poor housing taking priority over health
Anything else?
What do you think helps or could help Travellers' to seek help early?

Finally I've a couple of questions on accessing services …..
I'd like to ask you about Travellers' access to health care overall and then to cancer services … what are your experiences?
have times to get an appointment taken longer because of Covid?
What are your experiences of accessing services, please tell me about this?
Final Q. Is there anything you would like to add that you think will help Travellers' to prevent cancer and get diagnosed sooner…… (if we haven't already mentioned it)

Thank you very much for taking part in this interview.
