# Supplementary material for: Irish Travellers' Cancer Awareness and Engagement With Prevention, Screening and Early Diagnosis: A Qualitative Study of Barriers and Enablers
Source: Health Expect. 2026 Sep 21;29(5):e70866. doi: 10.1111/hex.70866 (PMC13594775; doi:10.1111/hex.70866)
Supplement: Supplementary file 3 [file HEX-29-e70866-s003.pdf]

Public Involvement Case Study Collection

(Based on GRIPP 2 Short Form Version)

Project/study name	Irish Travellers' Cancer Awareness and Engagement with Prevention, Screening and Early Diagnosis: A Qualitative Study of Barriers and Enablers
Researcher lead name	Removed for peer review PF
PPIE lead/co-ordinator name	MBC and LK
PPIE Groups utilised (if relevant)	
Number of public contributors used in the project	MBC and LK
Diversity of public contributors used in the project	Minority ethnic community. Pavee Point Traveller and Roma Centre

**Section**	**Description of section**	**Answers**
Aim	Report the aim of PPI in the study	3 to 6
Methods	Provide a clear description of the methods used for PPIE in the study	3 to 6
Study results	Outcomes – Report the results of PPI in the study, including both positive and negative outcomes	6 to 15
Discussion and conclusions	Outcomes – Comment on the extent to which PPI influenced the study	16to 20

	overall. Describe positive and negative effects	
Reflections/critical perspective	Comment critically on the study, reflecting on the things that went well and those that didn't, so others can learn from this experience	16- 20
